# Bioinformatic Analysis Identifies Three Potentially Key Differentially Expressed Genes in Peripheral Blood Mononuclear Cells of Patients with Takayasu’s Arteritis 

**DOI:** 10.22074/cellj.2018.4991

**Published:** 2017-11-04

**Authors:** Renping Huang, Yang He, Bei Sun, Bing Liu

**Affiliations:** 1 Department of General Surgery, The First Affiliated Hospital of Harbin Medical University, Harbin, China; 2 Department of Anesthesiology, The First Affiliated Hospital of Harbin Medical University, Harbin, China; 3 Department of Pancreatic and Biliary Surgery, The First Affiliated Hospital of Harbin Medical University, Harbin, China

**Keywords:** Candidate Gene, Peripheral Blood Mononuclear Cell, Protein-Protein Interaction Network, Takayasu’s Arteritis

## Abstract

**Objective:**

This study aimed to identify several potentially key genes associated with the pathogenesis of Takayasu’s arteritis (TA). This identification may lead to a deeper mechanistic understanding of TA etiology and pave the way for potential therapeutic approaches.

**Materials and Methods:**

In this experimental study, the microarray dataset GSE33910, which includes expression data for peripheral blood mononuclear cell (PBMC) samples isolated from TA patients and normal volunteers, was downloaded from the publicly accessible Gene Expression Omnibus (GEO) database. Differentially expressed genes (DEGs) were identified in PBMCs of TA patients compared with normal controls. Gene ontology (GO) enrichment analysis of DEGs and analysis of protein-protein interaction (PPI) network were carried out. Several hub proteins were extracted from the PPI network based on node degrees and random walk algorithm. Additionally, transcription factors (TFs) were predicted and the corresponding regulatory network was constructed.

**Results:**

A total of 932 DEGs (372 up- and 560 down-regulated genes) were identified in PBMCs from TA patients. Interestingly, up-regulated and down-regulated genes were involved in different GO terms and pathways. A PPI network of proteins encoded by DEGs was constructed and RHOA, FOS, EGR1, and GNB1 were considered to be hub proteins with both higher random walk score and node degree. A total of 13 TFs were predicted to be differentially expressed. A total of 49 DEGs had been reported to be associated with TA in the Comparative Toxicogenomics Database (CTD). The only TA marker gene in the CTD database was *NOS2*, confirmed by three studies. However, *NOS2* was not significantly altered in the analyzed microarray dataset. Nevertheless,*NOS3* was a significantly down-regulated gene and was involved in the platelet activation pathway.

**Conclusion:**

*RHOA, FOS*, and *EGR1* are potential candidate genes for the diagnosis and therapy of TA.

## Introduction

Takayasu’s arteritis (TA), also known as aortoarteritis and pulseless disease, is a rare chronic large vessel vasculitis of unknown etiology, affecting large arteries, especially the aorta and its main branches and the pulmonary artery (1). TA can lead to progressive occlusion, stenosis or aneurismal transformation (2). It usually affects young women under 40 years of age and is associated with important morbidity and mortality, particularly if there is a diagnostic delay (3). Therefore, gaining more insight into the underlying mechanism of TA is of great significance.

Clinically, TA includes an early phase with nonspecific systemic signs and symptoms of fever, arthralgia, night sweats, headaches, weight loss and myalgia (4). As vessel inflammation progresses, clinical features of this disease become apparent due to wall thickening, fibrosis, stenosis and thrombus formation (5). Tripathy et al. (6) investigated the expression of different cytokines at the transcript level in peripheral blood mononuclear cells (PBMCs) of patients with TA and demonstrated an inflammatory cytokine signature in TA with key roles suggested for tumor necrosis factor (TNF)-α, interleukin (IL)-4, and IL-10 in different pathological processes of this disorder. In addition, Soto et al. (7) identified the presence of *IS6110* and HupB gene sequences associated with *Mycobacterium tuberculosis* in aortic tissues of TA patients. Recently, a study identified a novel susceptibility locus in the IL12B region for TA which could be potentially used as a genetic marker for the severity of this disease (8). However, data on genes associated with TA are very limited. The identification of key genes associated with this disorder may provide insight into the pathogenesis underlying TA and provide novel avenues for therapeutic intervention in TA.

Okuzaki et al. (9) used high-density oligonucleotide microarrays to identify genes expressed in PBMCs of TA patients regardless of symptoms and demonstrated that Ficolin 1 (*FCN1*) expression was elevated in peripheral blood samples of TA patients. In this study, we downloaded and reanalyzed this microarray dataset and differentially expressed genes (DEGs) were identified in PBMCs of TA patients compared with normal controls. Gene ontology (GO) enrichment analysis of DEGs and analysis of their interactions in the protein-protein interaction (PPI) network were carried out. Several hub proteins were extracted from the PPI network based on node degree and random walk algorithm. Additionally, transcription factors (TFs) were predicted and the regulatory network was constructed. We sought to identify several potentially key genes associated with the pathogenesis of TA, which may lead to a deeper mechanistic understanding of TA and potential therapeutic approaches.

## Materials and Methods

In this experimental study, the gene expression dataset GSE33910 deposited by Okuzaki et al. (9) was downloaded from the publicly accessible Gene Expression Omnibus (GEO) database (http://www.ncbi. nlm.nih.gov/geo/) (10). Expression data were generated on a GPL4133 Agilent-014850 Whole Human Genome Microarray 4x44K G4112F. A total of 8 two-color arrays were included and each individual array comprised a target sample (Cy5-labeled) and a control sample (Cy3- labeled). The target samples were derived from PBMCs isolated from eight TA patients who were diagnosed as TA. All RNA samples isolated from the PBMCs of 17 healthy normal volunteers were included as control samples.

### Data preprocessing and screening of differentially expressed genes

The raw data in TXT format were downloaded. The two-color array data was preprocessed by using the Bioconductor limma package (11), including background correction, normalization and expression value calculation (12). Subsequently, by using the limma package (11), we identified DEGs between TA samples and control samples. The P values of an unpaired Student’s t test (13) with the matrix of gene expression values as the response variable was used to evaluate significance of differential expression. The Benjamini-corrected false discovery rate (FDR) (14) was applied to correct the raw P values for multiple testing. To get a reliable list of significant DEGs, the results were concurrently filtered using a cutoff of |log_2_FC (fold change)| and FDR-corrected P values. The selection of a specific threshold in this study was based on the usefulness of the data at hand and the results obtained for the follow-up analyses. Thus, cutoff points for up- and down-regulated genes in this study were |log_2_FC| ≥1.5 and FDR<0.05.

### Functional and pathway enrichment analysis

To interpret biological function of the DEGs, we performed an enrichment analysis in terms of GO and the Kyoto encyclopedia of genes and genomes (KEGG) pathways. GO term and KEGG pathway analyses were carried out with the web-based tool DAVID bioinformatics resources (version 6.8) (15). The selection of a cut-off criterion for GO and pathway enrichment analysis was based on the number of observed GO terms or pathways. In this study, P<0.05 (by hypergeometric test) were used as an empirical threshold for retrieving altered pathways or GO terms with gene count ≥2.

### Construction of protein-protein interaction network and extraction of significant nodes

The STRING database (http://string-db.org) provides a critical integration of PPIs, including known and predicted interactions. The interaction evidence could be examined by adjusting the interaction confidence scores, which is a key feature of STRING (16). Furthermore, each interaction in the STRING database is annotated with benchmarked confidence scores (low confidence: scores <0.4, medium: 0.4 to 0.7, high: >0.7) (17). In this study, a PPI network of proteins encoded by DEGs was constructed using the STRING database. The PPI score was set at 0.7 (high confidence). Additionally, network visualization was performed with the Cytoscape software (18). Nodes in the PPI network represented proteins and the interaction between any two nodes is represented by an edge. The random walk algorithm on the network is an iterative walker’s transition from a given seed node (protein) to a randomly selected neighbor based on the structure of the network (19). In each step, the walk has a probability of returning to the initial nodes. Finally, each node (protein) in the random walk process can be assigned with a transition probability which is proportional to the frequency of the interactions between the seed proteins and other proteins (19, 20). Genes with higher random walk score were considered to be significant nodes in the network. In this study, we employed a random walk algorithm for the genes in the PPI network to prioritize significant genes using the R package RWOAG (https://r-forge.r-project.org/ R/?group_id=1126) (19). Additionally, significant genes were also identified by degree centrality.

### Transcription factor prediction and construction of the regulatory network

iRegulon, a Cytoscape plugin, implements a genome-wide ranking-and-recovery approach and relies on the analysis of the regulatory sequences flanking each gene to detect enriched TF motifs and their optimal direct target subsets (21). We therefore used the iRegulon plugin (21) to identify TFs potentially regulating the DEGs and those of which were differentially expressed. The parameters were set at minimum identity between orthologous genes: 0.05 and maximum FDR on motif similarity: 0.001. The normalized enrichment score (NES)>3 was considered as a cut-off for the selection of the predicted TFs and their targets. The threshold value for differentially expressed TFs was |log_2_FC|≥0.5 and P<0.05, which is less stringent than that for DEG screening.

### Takayasu’s arteritis-associated gene prediction

The comparative toxicogenomics database (CTD, http:// ctdbase.org/) is a public resource that provides information on connections between environmental chemicals or drugs and gene products, and their relationships to different disorders (22). This databased was used to identify DEGs that have been reported to be associated with TA in the literature.

## Results

### Differentially expressed genes identification and functional enrichment analysis

The expression profile of TA pathogenesis was explored by identifying DEGs in PBMC samples of TA patients compared with normal controls. Of the total of 19, 215 genes analyzed on the microarray, 932 genes were differentially expressed of which 372 were up-regulated and 560 were down-regulated. To investigate which cellular functions were affected by these DEGs, GO terms and pathway enrichment analysis was conducted. The top five over-represented GO terms under three cellular categories [biological process (BP), cellular component (CC), and molecular function (MF)] and the main enriched pathways are shown in Table 1. Interestingly, we found that the up-regulated genes and the down-regulated genes were involved in different GO terms and pathways.

**Table 1:** Significant pathways and top five GO terms enriched by DEGs


Expression change	Category	Term	Count	P value

**Up**	BP	GO:0010467~gene expression	40	1.23E-05
		GO:0060968~regulation of gene silencing	5	4.30E-05
		GO:0007596~blood coagulation	23	2.99E-04
		GO:0038061~NIK/NF-kappaB signaling	8	3.33E-04
		GO:0006521~regulation of cellular amino acid metabolic process	7	4.66E-04
	CC	GO:0070062~extracellular exosome	105	3.38E-12
		GO:0005829~cytosol	97	3.81E-06
		GO:0005654~nucleoplasm	76	1.16E-04
		GO:0005839~proteasome core complex	5	4.22E-04
		GO:0000502~proteasome complex	7	9.47E-04
	MF	GO:0005515~protein binding	206	7.95E-05
		GO:0004298~threonine-type endopeptidase activity	5	6.73E-04
		GO:0044822~poly(A) RNA binding	35	7.68E-03
		GO:0005102~receptor binding	14	1.98E-02
		GO:0003924~GTPase activity	10	3.03E-02
	PATHWAY	hsa03050:Proteasome	7	4.85E-04
		hsa05133:Pertussis	7	7.67E-03
		hsa05034:Alcoholism	10	2.17E-02
		hsa00760:Nicotinate and nicotinamide metabolism	4	2.89E-02
		hsa00240:Pyrimidine metabolism	7	3.49E-02
		hsa05322:Systemic lupus erythematosus	8	3.60E-02
**Down**	BP	GO:0006415~translational termination	11	4.15E-05
		GO:0016259~selenocysteine metabolic process	11	5.60E-05
		GO:0001887~selenium compound metabolic process	12	1.01E-04
		GO:0006414~translational elongation	11	1.29E-04
		GO:0006614~SRP-dependent cotranslational protein targeting to membrane	11	2.89E-04
	CC	GO:0001533~cornified envelope	7	8.47E-04
		GO:0030141~secretory granule	8	2.37E-03
		GO:0022627~cytosolic small ribosomal subunit	6	3.11E-03
		GO:0022625~cytosolic large ribosomal subunit	6	1.45E-02
		GO:0005615~extracellular space	42	4.72E-02
	MF	GO:0003735~structural constituent of ribosome	13	3.94E-03
		GO:0043565~sequence-specific DNA binding	18	1.37E-02
		GO:0005179~hormone activity	7	2.39E-02
		GO:0005198~structural molecule activity	11	3.34E-02
		GO:0005201~extracellular matrix structural constituent	5	3.50E-02
	PATHWAY	hsa03010:Ribosome	13	6.69E-05
		hsa04080:Neuroactive ligand-receptor interaction	14	1.32E-02
		hsa04721:Synaptic vesicle cycle	6	1.59E-02
		hsa04724:Glutamatergic synapse	8	1.76E-02
		hsa04010:MAPK signaling pathway	12	3.66E-02


Go; Gene ontology, DEGs; Differentially expressed genes, BP; Biological process, CC; Cellular component, and MF; Molecular function. P values were calculated by the hypergeometric test.

### Protein-protein interaction network construction and prediction of significant genes

**Fig.1:**
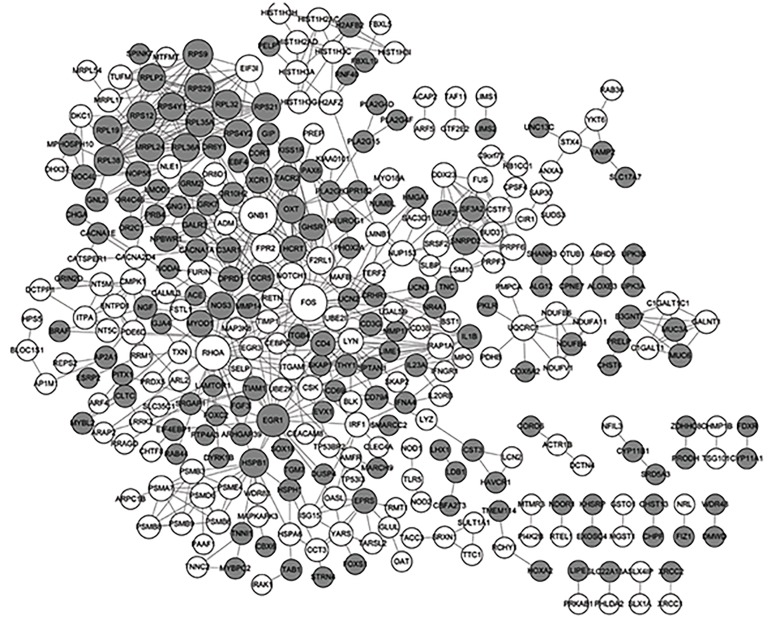
Visual representation of the protein-protein interaction (PPI) network. The white nodes represent up-regulated genes and the gray nodes indicate down-regulated genes. The size of the nodes was positively correlated with node degree.

The PPI network consisted of 627 interactions (edges) among 330 proteins (nodes) (Fig .1). The top 15 DEGs in the network ranked by the random walk score and top DEGs ranked by degree are shown (Table 2). The top four genes in the network based on random walk score and node degree were Ras homolog family member A (*RHOA*, up-regulated), FBJ murine osteosarcoma viral oncogene homolog (*FOS*, up-regulated), early growth response 1 (*EGR1*, down-regulated), and G protein subunit beta 1 (*GNB1*, up-regulated). On the other hand, nitric oxide synthase 3 (*NOS3*, downregulated) ranked 6 based on the random walk score, while the node degree of *NOS3* was 11. Besides, a number of RPL family members were with a higher node degree but were not in the top 15 nodes ranked by the random walk score. These results suggested that there was little difference in the list of significant genes in the PPI network identified by the random walk algorithm with that based on node degree. In addition, we performed pathway enrichment analysis on the top 15 DEGs. The results showed that these DEGs were predominantly involved in 4 pathways, namely hsa05133:pertussis, hsa04921:oxytocin signaling pathway, hsa04611:platelet activation, and hsa04062:chemokine signaling pathway. Interestingly, *RHOA, NOS3*, and LYN Proto-Oncogene, Src Family Tyrosine Kinase (*LYN*) were involved in platelet activation, a TA-associated pathway.

**Table 2:** The top 15 DEGs ranked by the random walk score and their degree


Node	Random walk score	Node	Degree

*RHOA*	0.0084665	*GNB1*	27
*FOS*	0.0078574	*FOS*	26
*EGR1*	0.0074923	*RHOA*	23
*GNB1*	0.0062084	*EGR1*	19
*NOS3*	0.00601	*RPL38, RPS9, RPS29*	15
*UQCRC1*	0.0058351	*RPS12, FPR2, RPL32, RPL19*	14
*MYOD1*	0.0054857	*HSPB1, MRPL24, RPL35A, RPS21*	13
*HSPB1*	0.0052129	*OXT, RPS4Y2, RPS4Y1, RPLP2, RPL36A*	12
*NOTCH1*	0.0048462	*NOS3, SNRPD2, F2RL1, EIF3I*	11
*LYN*	0.0047398	*MYOD1, NOTCH1, GHSR*	10
*IRF1*	0.004669	*RAP1A, SRSF2, PRPF6, CCR5, XCR1, HCRT*	9
*ITGAM*	0.0044762	*UQCRC1, LYN, ITGAM, NUP153, PSMD6, PSMA7, CMPK1, CSTF1, FUS, SF3A2, U2AF2, TACR2, KISS1R*	8
*EPRS*	0.004437	*IRF1, EPRS, UBE2I, CD4, CSK, NOP58, PSMB3, DDX23, GALR3, GRM2, C3AR1, NPBWR1, OPRD1*	7
*STX4*	0.0042952	*H2AFZ, YARS, TIAM1, IL1B, LMNB1, NOC4L, PDE6D, TUFM, ENTPD1, MMP14, PSMB6*	6
*OXT*	0.0042259	*FURIN, CCT3, HIST1H3A, TIMP1, B3GNT7, HIST1H2AC, HIST1H2AD, H2AFB2, CD79A, NGF, MRPL17, GALNT1, MUC3A, MUC6, C1GALT1C1, NT5C, NT5M, OASL, ISG15, EGR3, ITPA, PSMB8, OR4C46, OR8D1, OR2C1, OR6Y1, OR10H2, NLE1, PSME4, CD3G*	5


### Transcriptional regulation network analysis and prediction of Takayasu’s arteritis-associated genes

**Fig.2:**
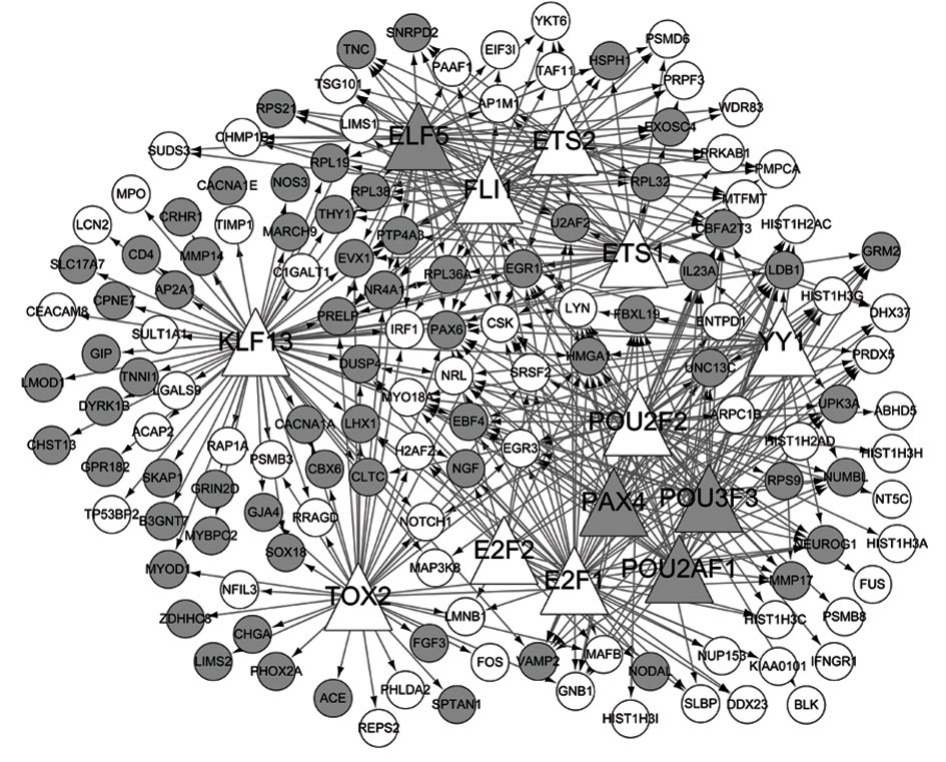
Visual representation of the transcriptional regulatory network. The white nodes represent up-regulated genes and the gray nodes indicate down-regulated genes. Triangular nodes indicate transcription factors (TFs) and round nodes denote target genes.

A total of 13 TFs were predicted to be differentially expressed (Table 3), such as the YY1 Transcription Factor (YY1) and the Fli-1 Proto-Oncogene, ETS Transcription Factor (FLI1). The transcriptional regulatory network was constructed (Fig .2). Based on the CTD database, a total of 893 genes had curated or inferred association with TA of which 49 were identified to be DEGs in this study (Table 4). The only TA marker gene in the CTD database was *NOS2*, confirmed by three studies (23-25). However, *NOS2* was not significantly altered in the analyzed microarray dataset. Nevertheless, NOS3 was a significantly down-regulated gene and was also a hub in the PPI network.

**Table 3:** Expression profile of predicted TFs


TF	Log FC	P.value	adj.P.val

*POU2F2*	1.322403	2.06E-02	0.081647
*ETS2*	1.322031	2.15E-02	0.078272
*YY1*	1.264822	1.11E-03	0.019872
*FLI1*	1.22772	4.01E-03	0.034229
*TOX2*	1.062465	1.08E-03	0.021665
*E2F2*	1.052923	9.83E-03	0.04705
*E2F1*	0.96085	3.17E-03	0.031772
*KLF13*	0.71519	3.47E-02	0.108853
*ETS1*	0.563542	2.51E-02	0.072435
*POU3F3*	-0.96573	1.39E-02	0.063961
*POU2AF1*	-0.96704	9.49E-03	0.051347
*ELF5*	-1.0965	1.42E-02	0.05923
*PAX4*	-2.0087	3.69E-02	0.101316


TF; Transcription factor.

**Table 4:** DEGs associated with TA in the CTD database


Expression change	TA gene

Up	*DNPH1, ILVBL, CRAT, TNFSF13, ARAP2, PGD, IMPA2, TIMM10B, PSMB9, PSMB8, DCTPP1, PSMB3, SRXN1, OAT, GBA, CSK, RCHY1, LYN, SRSF2, IRAK1, AUP1, TMEM60, LSM10, PSMB6, COASY, PRKAB1, HAL, KYAT1, GADD45A, NEU1, RCSD1*
Down	*C3AR1, PVT1, EXOSC4, CPNE1, EXOC3L2, HSPH1, ABHD4, HSPB1, RPS9, LENG8, SERPINA6, PKLR, MYOD1, IL1B, REEP6, THEM5, MECR, RAB30*


TA; Takayasu’s arteritis and CTD; Comparative toxicogenomics database.

## Discussion

In the present study, by integrating the expression profile of TA patients, we identified three potentially key genes in TA, namely *RHOA, FOS,* and *EGR1. RHOA* encodes a member of the Rho family of small GTPases and functions as a molecular switch in signal transduction cascades (26). Ample evidence has demonstrated that *RHOA* is required for transendothelial migration, a tightly regulated process whereby leukocytes migrate from the vasculature into tissues (27-29). Transendothelial migration of leukocytes to the sites of inflammation is a critical step in the inflammatory response (29). Moreover, TA is a chronic disease characterized by inflammation of large vessels (30). In this study, *RHOA* was not only a hub protein in the PPI network, but it also was upregulated. These results suggest that *RHOA* may play a key role in the pathogenesis of TA. Experimental validation will be needed to confirm this finding.

*FOS* was also found to be a hub in the PPI network of DEGs. FOS encodes a leucine zipper protein which can dimerize with proteins of the JUN family, thereby forming the transcription factor complex AP-1 (31). AP-1 can in turn be phosphorylated and regulated by mitogen-activated protein kinases (MAPKs) in response to Toll-like receptor (TLR) signaling stimuli (32). Additionally, a study has shown that TLR ligands can function as instigators of vessel wall inflammation in giant cell arteritis, another type of large vessel vasculitis (33). Overall, our results are consistent with the accumulating evidence implicating FOS in the pathogenesis of TA through the TLR signaling pathway.

The third hub based on both higher random walk score and node degree was *EGR1*. The protein encoded by *EGR1* belongs to the EGR family of C2H2-type zincfinger proteins. It is a nuclear protein and functions as a transcriptional regulator (36). Decker et al. (35) showed that EGR-1 could regulate IL-2 transcription by a synergistic interaction with the nuclear factor of activated T cells. In addition, Tripathy et al. (36) demonstrated that high TNF-α and low IL-2-producing T cells played an important role in TA. This is consistent with the observation of *EGR-1* being a down-regulated hub. Thus, we suggest that down-regulation of *EGR-1* in PBMCs of TA patients may be pathogenically significant. However, further investigations are needed to validate this association.

We conclude that the several putative genes identified here, in particular *RHOA, FOS* and *EGR1*, may play key roles in the pathogenesis of TA and could become potential targets for future therapeutic strategies. The limitation of this analysis was the small sample size in this study and lack of experimental validation. Further studies with larger sample size concerning these genes may confirm the hitherto unknown mechanism underlying the pathogenesis of TA. This in turn may expand the therapeutic arsenal against TA.
